# Identification of Amino Acid Propensities That Are Strong Determinants of Linear B-cell Epitope Using Neural Networks

**DOI:** 10.1371/journal.pone.0030617

**Published:** 2012-02-08

**Authors:** Chun-Hung Su, Nikhil R. Pal, Ken-Li Lin, I-Fang Chung

**Affiliations:** 1 Institute of Biomedical Informatics, National Yang-Ming University, Taipei, Taiwan, Republic of China; 2 Electronics and Communication Sciences Unit, Indian Statistical Institute, Calcutta, India; 3 Computer Center, Chung Hua University, Hsinchu,Taiwan, Republic of China; Semmelweis University, Hungary

## Abstract

**Background:**

Identification of amino acid propensities that are strong determinants of linear B-cell epitope is very important to enrich our knowledge about epitopes. This can also help to obtain better epitope prediction. Typical linear B-cell epitope prediction methods combine various propensities in different ways to improve prediction accuracies. However, fewer but better features may yield better prediction. Moreover, for a propensity, when the sequence length is *k*, there will be *k* values, which should be treated as a single unit for feature selection and hence usual feature selection method will not work. Here we use a novel Group Feature Selecting Multilayered Perceptron, GFSMLP, which treats a group of related information as a single entity and selects useful propensities related to linear B-cell epitopes, and uses them to predict epitopes.

**Methodology/ Principal Findings:**

We use eight widely known propensities and four data sets. We use GFSMLP to rank propensities by the frequency with which they are selected. We find that Chou's beta-turn and Ponnuswamy's polarity are better features for prediction of linear B-cell epitope. We examine the individual and combined discriminating power of the selected propensities and analyze the correlation between paired propensities. Our results show that the selected propensities are indeed good features, which also cooperate with other propensities to *enhance* the discriminating power for predicting epitopes. We find that individually polarity is not the best predictor, but it *collaborates* with others to yield good prediction. Usual feature selection methods cannot provide such information.

**Conclusions/ Significance:**

Our results confirm the effectiveness of active (group) feature selection by GFSMLP over the traditional passive approaches of evaluating various combinations of propensities. The GFSMLP-based feature selection can be extended to more than 500 remaining propensities to enhance our biological knowledge about epitopes and to obtain better prediction. A graphical-user-interface version of GFSMLP is available at: http://bio.classcloud.org/GFSMLP/.

## Introduction

B-cell epitopes are antigenic determinants, which are recognized and bound by B-cell receptors or antibodies [1]. Knowledge of the locations of B-cell epitopes can help develop peptide vaccines or can be used to induce the production of antibodies that can be applied as diagnostic or therapeutic tools in the laboratory or by pharmaceutical industry [2]–[4]. There are two kinds of B-cell epitopes: Linear B-cell epitopes and conformational B-cell epitopes. Linear B-cell epitopes are constructed from contiguous residues from the amino acid sequence of a protein and the conformational B-cell epitopes are formed by non-contiguous residues which become adjacent as a result of folding of a protein structure [5]. Many studies have reported success of sequence-based prediction approaches for different biological problems, such as prediction of protein pathway networks [6], protein subcellular location [7], [8], and drug-target interaction [9]. There are other sequence-based methods for identification of membrane proteins and their types [10], prediction of the metabolic stability of proteins [11], identification of enzymes and their functional classes [12], prediction of network of substrate-enzyme-product triads [13], identification of GPCR and their types [14], and identification of proteases along with their types [15]. These sequence-based prediction methods as well as some of the user-friendly web-servers for predicting various attributes of proteins are recently summarized in [16]. In this study, we try to develop a novel sequence-based method for identification of amino acid propensities that are strong determinants of epitopes. We also investigate the effectiveness of those selected propensities in epitope prediction. Since in wet-lab operations contiguous peptide sequences are more easily synthesized, many studies for B-cell epitope identification have focused on prediction of linear B-cell epitopes. Here we focus only on linear B-cell epitope prediction.

In the past three decades, many studies attempted to predict the locations of linear B-cell epitopes in a protein sequence. Generally, those studies can be classified as sliding-window-based or machine-learning-based approaches. The sliding-window-based approaches assume that the locations of linear B-cell epitopes are highly correlated to certain physico-chemical properties. Such a method considers some propensity value (say hydrophilicity) of amino acids and computes the average value of this propensity measure over a window of fixed length in a protein sequence [17]. For example, if the window length is *w* = 2*k*+1, then sliding of the window starts from the left end. The first average value corresponds to the residue location *k*+1. Then the window is shifted one position to the right and again the average is computed which corresponds to residue location *k*+2. The process is continued till the window reaches the end of the sequence. Then residues with average value greater than a threshold are labelled as possible linear epitopes. Some methodologies use more than one propensity values which are combined using different weighting factors. The literature is quite rich in this area [18]–[27]. However, Blythe and Flower demonstrated after exhaustive testing that a single amino acid propensity scale may not be effective to predict epitope location reliably [28]. And they suggested that artificial intelligence techniques would be better to improve the prediction performance. Furthermore, the weak predictive performance, irrespective of whether we use single or multiple propensities, could result from the fact that these average values may be above the threshold in one segment and less than the threshold in the *next* segment. In this case the two segments will have (*w*−1) common residues, but one residue will be labelled as an epitope and the other will not be!

Hence, after the first B-cell epitope database [29] was carefully curated for linear epitopes, there have been many attempts using machine learning approaches to improve the prediction of the locations of linear B-cell epitopes [30]–[35]. However, for machine learning approaches, use of different combinations of features is known to result in different prediction performance. More features are not necessarily better. On the other hand, use of a smaller set of useful features can enhance the prediction accuracy, particularly for the test data. In addition use of less features leads to lesser degrees of freedom of the trained system and hence chances of memorization of data would be lower. Therefore, selection of an appropriate set of features (could be propensities) without using an exhaustive search for the prediction of linear B-cell epitopes is an important problem to address.

Generally, feature selection methods fall into two broad groups, namely, Filter method and Wrapper method [36], [37]. The filter method does not take any feedback from the classifier or the predictor that will ultimately use the selected features. A wrapper method, evaluates the effectiveness of the features using the classifier (or prediction system) that will finally use the selected features. The Wrapper method, thus, are likely to exhibit better performance. For many learning problems, features may have natural grouping and either a group as a whole should be selected or discarded. As an example [38], consider an intelligent weld inspection system for which the sources of information may be X-ray images, radiographs, eddy current and so on. Here we cannot use an X-ray image directly into a pattern recognition system, but first a set of features has to be computed from it and then that set of features can be used. The same is true for radiographs. Thus, for a real-time intelligent weld inspection system, we need to minimize the number of sensors, which will reduce the size, design cost and processing cost associated with the system. So either we discard entirely an X-ray image or accept it. Similarly, in bioinformatics, we may compute a set of features from a propensity measure. So discarding a propensity measure results in discarding the set of features computed from the propensity measure. This is selection of subsets of features and it is a generalized form of feature selection. To address this problem, in our earlier study we have proposed two integrated methods, the Group Feature Selecting Multilayered Perceptron (GFSMLP) and the Group Feature Selection RBF (GFSRBF) network, which can select/discard subsets of features [38] – to our knowledge this is the first work in this area. Here we shall use only the GFSMLP network to find useful amino acid propensities for linear B-cell epitope prediction. Note that, in previous linear B-cell epitope prediction studies, researchers have only focused on how to achieve a better epitope prediction using various combinations of amino acid propensities as input features to a classifier, without actually performing the feature selection.

In this study we use GFSMLP technique on four data sets considering eight widely used amino acid propensities, to determine which, and to what extent, certain amino acid propensities are better at working together to solve the linear B-cell epitope prediction problem. In addition, we also perform some validation experiments to examine whether the selected amino acid propensities are reasonable with respect to all four data sets. Also, in order to offer a user-friendly solution, we provide a graphical user interface (GUI) version of our GFSMLP program (http://bio.classcloud.org/GFSMLP/) so that users with no proficiency in programming can simply go all the way from uploading their data set to selecting useful features and obtaining relevant results.

## Results and Discussion

In this study we use GFSMLP to identify amino acid propensities that are good determinants of linear B-cell epitopes or non-B-cell epitopes. Our approach can find the propensities that interact linearly or non-linearly to determine the location of linear B-cell epitopes. Here we have performed two experiments on four data sets to show that our approach not only identifies amino acid propensities which are good discriminators individually but also groups of propensities that cooperate for better prediction of epitopes. In particular, we have identified some *specific* pair of propensities that are good determinants of epitopes. In addition, two classification procedures, GFSMLP and a two-level 10-fold cross-validation scheme with Support Vector Machine (SVM) classifier [39] are utilized to assess the discriminating power of a selected propensity or pair of propensities. Finally, for each data set we also examine the correlations of paired propensities to further understand why two specific propensities cooperate well. Our results are divided into three subsections: ranking of amino acid propensities, understanding cooperation between propensities to determine linear B-cell epitopes, and correlations of paired propensities.

### Ranking of Amino Acid Propensities

The main objective here is to rank some of the amino acid propensities [19], [20], [22]–[24], [40]–[42] in terms of its relevance for prediction of linear B-cell epitopes. We want to find if one or more of the amino acid propensities tends to cooperate with other amino acid propensities in solving the linear B-cell epitope prediction problem well. Thus, in our first experiment, we conduct 1,000 runs of GFSMLP to get the ranking by the frequency with which amino acid propensities are selected (i.e., a propensity with a higher frequency is considered a better candidate). GFSMLP learning needs four parameters to be specified. For each run, in this experiment, we use the following values for the parameters: learning coefficients *μ* = 0.1 and *η* = 0.2, number of hidden units, *n* = 15, and number of iterations = 2,000. All issues relating to the optimal choice of parameters are ignored here as our goal is not to design the best classifier here. The training starts assuming all propensities as bad (assume that a gate is associated with every propensity and every gate is almost closed, see Materials and Methods). Table 1 summarizes the results. Table 1 reveals that Chou's beta-turn (propensity #8) [42] and Ponnuswamy's polarity (propensity #5) [41] are the most frequently selected propensities over 1,000 runs in all four data sets. Beta-turn is selected 629, 553, 554, and 643 times over the 1,000 runs for data sets AAP872, ABCpred, BCPred, and Combo, respectively. Polarity is selected 397, 307, 402, and 498 times over 1,000 runs in the same four data sets, respectively. On the other hand, Pellequer's turns (propensity #6) [24] and Janin's surface exposed scales (propensity #4) [40] are the least selected propensities for most of these four data sets. This suggests that Chou's beta-turn and Ponnuswamy's polarity, individually or together, are *probably* the most important discriminators while Pellequer's turns and Janin's surface exposed scales are the least important attributes contributing to the location of linear B-cell epitopes. We say, probably because features can interact between themselves and a feature, when working alone, may not be a good discriminator but it can do a great job in cooperation with some other features. Hence, we further use *two* evaluation strategies to determine classification performance based on the selected propensities. We use two classifiers, GFSMLP and SVM. In addition to GFSMLP, we use SVM because it has been found to be very effective in predicting membrane protein type, protein subcellular location, HIV protease cleavage sites in protein etc. [43]–[49].

**Table 1:** The frequency with which each propensity is selected in 1,000 runs of GFSMLP (*η* = 0.2, *μ* = 0.1, *n* = 15, number of iterations = 2,000).

Data SetsPropensities	AAP872	ABCpred	BCPred	Combo
1. Hydrophilicity (Parker)	315	178	295	386
2. Accessibility (Emini)	232	214	251	315
3. Flexibility (Karplus)	239	162	216	305
4. Surface Exposed Scale (Janin)	149	183	186	250
5. Polarity (Ponnuswamy)	397	307	402	498
6. Turns (Pellequer)	186	147	91	209
7. Antigenicity (Kolaskar)	240	206	140	309
8. Beta-turn (Chou)	629	553	554	643

In the first evaluation experiment we use GFSMLP to verify the utility of the selected propensities. Here we proceed as follows. We use all of the data to construct and test the classifier (GFSMLP) to determine which propensity is better for the linear B-cell epitope prediction. Note that, our intention is not to design a classifier but to find which propensities are more useful for this problem of linear B-cell epitope prediction. For this set of experiments, the parameters for GFSMLP are set as *μ* = 0, *η* = 0.2, *n* = 15, number of iterations = 2,000 and the gate associated with the selected propensity is kept completely open while for all other propensities the gates are completely closed. Since, *μ* = 0, so no training of the attenuators will be done. We repeat such experiments 100 times. In Table 2 we report the average misclassification rates and standard deviations. These are training errors and not to be confused with test error. The first column shows the propensity whose gate is kept open (i.e., the propensity that is used). Table 2 brings out a few interesting points: (a) GFSMLP suggested Chou's beta-turn (propensity #8) as the most useful predictor for linear B-cell epitopes and Table 2 reveals that it is indeed the case. It demonstrates that this propensity has the best discrimination power *by itself* in solving the linear B-cell epitope prediction problem in all four data sets. Thus, as of this point in our analysis, we can infer that Chou's beta-turn propensity is the best choice to serve as one of the input features for linear B-cell epitope prediction. It also has the best tendency to cooperate with other propensities. (b) Previously we have found that Ponnuswamy's polarity is the second most frequently selected propensity by GFSMLP. But here we find that, this propensity alone is not the second best predictor; in fact, it is the second worst predictor when considered alone. Hence, this propensity alone may not be a good feature for a classifier but it may play a good supporting role to obtain a better linear B-cell epitope prediction performance when cooperating with other propensities. We shall demonstrate this later. (c) When we look at the performance of Pellequer's turns (Propensity #6) and Janin's surface exposed scales (Propensity #4), we find that none of them alone has good prediction ability. Both of them have quite poor discrimination power *by themselves*. The propensity #6 is the *worst* in all cases but ABCPred data set, where it was the second worst. The propensity #4 is the *third* worst for every data set in the linear B-cell epitope prediction. It suggests that none of these propensities, when used alone, is a good feature for the classification of linear B-cell epitopes. (d) The other propensities appear to have reasonable discriminating power when used individually, but may not easily cooperate with other propensities for linear B-cell epitope prediction. For example, Emini's accessibility (Propensity #2), although exhibits a good discriminating power alone (the second best), considering Table 1 we find that it does not easily cooperate with other propensities as it is selected less than 25% times in conjunction with other propensities. However, these identified poor propensities may perform well when used in conjunction with some other propensities not considered in this study. This is left as a subject for future study.

**Table 2:** Average of misclassification rates over 100 runs of GFSMLP using the selected propensity (*η* = 0.2, *μ* = 0, *n* = 15, number of iterations = 2,000).

Data setsPropensities	AAP872	ABCpred	BCPred	Combo
1. Hydrophilicity (Parker)	9.43±0.79	5.46±0.77	6.63±0.60	14.02±0.86
2. Accessibility (Emini)	8.87±0.70	4.79±0.48	6.09±0.49	13.24±0.74
3. Flexibility (Karplus)	10.01±0.84	5.34±0.66	6.99±0.74	14.50±1.03
4. Surface Exposed Scale (Janin)	15.80±2.67	6.72±1.05	8.68±1.05	20.31±1.93
5. Polarity (Ponnuswamy)	19.78±1.75	16.59±2.97	13.07±1.84	24.10±2.43
6. Turns (Pellequer)	23.49±2.25	11.24±2.08	16.25±2.64	26.37±2.06
7. Antigenicity (Kolaskar)	11.00±1.28	5.84±0.62	8.06±0.96	16.96±1.29
8. Beta-turn (Chou)	8.40±0.60	4.53±0.45	6.05±0.52	12.67±0.66

In the previous experiments we have evaluated propensities using a neural network (GFSMLP) and the ranking of propensities is also done using the same tool, GFSMLP. If Chou's beta-turn is indeed a good predictor of B-cell epitopes, then it should also do a good job of prediction using other classifiers such as SVM. This is what we test here. To get a reliable estimate of the prediction accuracy, here we adopt a two-level 10-fold cross-validation scheme with SVM. We use only one of the propensities as the feature. The resultant test accuracies are shown in Table 3, which also reveal several interesting phenomena: (a) here also we obtain similar accuracy (no more than 60%) as reported in previous studies using the cross-validation framework with any one of those eight propensities as input to the classifier [30], [31]. Although our results again demonstrate that the classification performance using an individual propensity is not as good as that using the frequencies of amino acid pairs in epitope/non-epitope peptides (about 10% improvement in accuracy) [31], our study opens up the possibility of using GFSMLP to identify other good features/propensities from the huge list of available propensities (currently 544 amino acid propensities can be obtained from http://www.genome.ad.jp/aaindex) [50] or from other characteristics of epitope/non-epitope peptides. (b) We obtain the same conclusions as in (a)–(d) of the previous paragraph, despite some minor deviations. For example, the identified good propensity, Chou's beta-turn, also has the best discrimination power *by itself* in solving the linear B-cell epitope prediction problem in most of the four data sets (e.g., in the data set AAP872, Chou's beta-turn has the third highest accuracy, but within a difference of a mere 0.29% from the best prediction accuracy). For specific results, see Table 3.

**Table 3:** The accuracy of SVM using just single propensity by the 2-level 10-fold cross validation scheme.

Data setsPropensities	AAP872	ABCpred	BCPred	Combo
1. Hydrophilicity (Parker)	56.99%	53.75%	58.00%	57.11%
2. Accessibility (Emini)	57 23%	56.10%	58.36%	57.52%
3. Flexibility (Karplus)	53.96%	54.23%	55.43%	56.10%
4. Surface Exposed Scale (Janin)	54.65%	55.29%	55.43%	57.48%
5. Polarity (Ponnuswamy)	52.76%	49.67%	52.86%	54.20%
6. Turns (Pellequer)	52.41%	52.59%	52.43%	52.67%
7. Antigenicity (Kolaskar)	55.50%	56.28%	55.29%	56.43%
8. Beta-turn (Chou)	56.94%	56.44%	58.64%	59.46%

### Do propensities interact to identify Epitopes?

In the previous experiments, we have considered only one property of amino acids. Here we want to check if pairs of propensities can interact to yield better prediction. We found Chou's beta-turn (propensity #8) as a good propensity for linear B-cell epitope prediction. Does it mean that beta-turn in conjunction with other propensity can produce better prediction. To answer this, we follow a similar (not same) approach as we did in the previous experiments. First we use GFSMLP and initially we make the gate completely open for a *particular* propensity and set the remaining gates almost closed. And then we train the network along with the gate modulators. The other parameters for GFSMLP remain the same as with the previous experiments. This GFSMLP experiment, with a particular gate open at the onset of training, is run 100 times and in each time we record the incidence of the selection of the remaining seven propensities.

In Table 4 we report the summary of these experiments. This table has four parts for the four data sets. First, it is interesting to note that the gate which is initialized to an open state continues to remain open suggesting that none of the considered propensities is a derogatory propensity for this problem. The last column in Table 4 reports the total number of times different propensities is selected when the gate corresponding to the propensity shown in the first column is set open at the onset of training. This total frequency is a good indicator of the ability of a specific propensity to work (collaborate) with other propensities. A careful inspection of Table 4 shows that propensity #5 (Ponnuswamy's polarity) is the most effective in collaborating with other propensities and it interacts strongly with Hydrophilicity (propensity #1), Flexibility (propensity #3), Turns (propensity #6), and Antigenicity (propensity #7) to predict location of linear B-Cell epitopes. Polarity also strongly cooperates with Beta-turn (propensity #8), which is the next most active one in terms of collaboration with other propensities. The propensity Beta-turn is most friendly with Surface Exposed Scale (propensity #4). On the other hand, Accessibility (propensity #2) interacts the least with other attributes for epitope prediction (it never selects Surface Exposed Scale for three of the data sets and is selected only twice for the combined data set). But from Table 2 we find that this propensity has a good discriminating power. Thus it suggest that Accessibility, although is a good discriminator, unlike Beta-turn, it cannot interact with others. Since Accessibility has a very strong correlation with Surface Exposed Scale (shown in Table S1 and explained in the next subsection), the behaviour of Surface Exposed Scale should be similar to Accessibility. Table 4 indeed reveals that Surface Exposed Scale does not collaborate with other attributes. These observations are consistent with Table 1. These observations about preferred propensities are quite consistent over different data sets.

**Table 4:** The frequency with which each feature/propensity is selected in 100 runs of GFSMLP (*η* = 0.2, *μ* = 0.1, *n* = 15, number of iterations = 2,000).

Propensities	[1]	[2]	[3]	[4]	[5]	[6]	[7]	[8]	Sum
**AAP872**									
[1] Hydrophilicity (Parker)	100	12	1	16	25	16	12	11	193
[2] Accessibility (Emini)	11	100	13	0	7	15	5	12	163
[3] Flexibility (Karplus)	4	12	100	13	13	17	12	22	193
[4] Surface Exposed Scale (Janin)	19	1	16	100	1	11	11	13	172
[5] Polarity (Ponnuswamy)	51	29	40	23	100	41	45	38	367
[6] Turns (Pellequer)	11	22	15	21	17	100	17	7	210
[7] Antigenicity (Kolaskar)	6	9	9	16	9	9	100	13	171
[8] Beta-turn (Chou)	22	44	25	49	23	25	27	100	315
**ABCpred**									
[1] Hydrophilicity (Parker)	100	4	0	14	10	11	4	1	144
[2] Accessibility (Emini)	2	100	2	0	1	10	3	4	122
[3] Flexibility (Karplus)	0	3	100	16	8	16	10	6	159
[4] Surface Exposed Scale (Janin)	8	0	5	100	0	11	6	9	139
[5] Polarity (Ponnuswamy)	30	18	27	13	100	31	33	20	272
[6] Turns (Pellequer)	15	9	8	16	4	100	12	2	166
[7] Antigenicity (Kolaskar)	4	2	2	5	2	19	100	3	137
[8] Beta-turn (Chou)	16	28	13	39	22	18	23	100	259
**BCPred**									
[1] Hydrophilicity (Parker)	100	7	1	12	18	21	9	3	171
[2] Accessibility (Emini)	4	100	8	0	5	18	7	9	151
[3] Flexibility (Karplus)	0	6	100	8	16	14	7	10	161
[4] Surface Exposed Scale (Janin)	8	1	9	100	1	13	10	13	155
[5] Polarity (Ponnuswamy)	41	32	43	19	100	33	39	33	340
[6] Turns (Pellequer)	16	15	12	19	9	100	18	1	190
[7] Antigenicity (Kolaskar)	6	4	2	10	14	12	100	11	159
[8] Beta-turn (Chou)	15	27	20	34	25	21	27	100	269
**Combo**									
[1] Hydrophilicity (Parker)	100	26	11	36	25	20	24	18	260
[2] Accessibility (Emini)	19	100	12	2	10	18	15	23	199
[3] Flexibility (Karplus)	4	12	100	22	30	19	18	16	221
[4] Surface Exposed Scale (Janin)	16	3	25	100	5	20	20	26	215
[5] Polarity (Ponnuswamy)	52	48	51	39	100	48	59	43	440
[6] Turns (Pellequer)	19	26	25	26	11	100	18	11	236
[7] Antigenicity (Kolaskar)	11	17	18	26	17	23	100	22	234
[8] Beta-turn (Chou)	35	53	29	52	39	34	47	100	389

We also observe in Table 4 that Flexibility (propensity #3) and Antigenicity (propensity #7) do not easily cooperate with propensity Hydrophilicity in all four data sets. Thus considering pairs of propensities we find that Ponnuswamy's polarity and Chou's beta-turn are better candidates to cooperate with other propensities in solving the B-cell epitope prediction problem. This is also consistent with Table 1. Also, we observe that Polarity, compared with Beta-turn, appears to be more versatile in its ability to collaborate more often with other propensities. We can make another interesting observation from Table 1 and Table 4. Table 1 suggests that propensity #6 (Pellequer's Turns) is the worst predictor of epitopes, but while considering its effect in conjunction with others we find that it has a good ability to interact with others in solving the epitope prediction problem. These are biologically interesting observations, which suggest what all attributes (properties of residues) determine the epitope/non-epitope nature. Note that, normal feature selection methods cannot provide this kind of biological insights.

Next we want to examine the discriminating power for every pair of propensities using SVM. Here also we use the same 10-fold cross validation mechanism. Table 5 records the performance using pairs of propensities. From this table we can make few interesting observations: (a) For all but AAP872 data set, the best as well as the second best performance are obtained using a pair involving propensity #8 (Chou's beta-turn). (b) In particular the propensity pair involving #2 and #8 yields the best performance for 2 of the four data sets. This reconfirms our inference that Chou's beta-turn is a better propensity to serve as one of the input features for linear B-cell epitope prediction. Chou's beta-turn has a better tendency to cooperate with other propensities. (c) Although Ponnuswamy's polarity, when used alone, has quite a low discriminating power, in conjunction with several other propensities, it can yield a good discriminating power. For example, in all data sets, propensity #1 when combined with propensity #5 produces better results than that using propensity #1 or propensity #5 alone.

**Table 5:** The accuracy of SVM using pair of propensities by 2-level 10-fold cross validation.

Data setsPropensities	AAP872	ABCpred	BCPred	Combo
Propensities: 1 & 2	58.49%	54.57%	59.93%	62.21%
Propensities: 1 & 3	56.42%	53.01%	56.43%	60.06%
Propensities: 1 & 4	57.05%	55.38%	59.57%	62.20%
Propensities: 1 & 5	58.43%	54.16%	59.07%	60.67%
Propensities: 1 & 6	57.28%	54.48%	57.57%	57.52%
Propensities: 1 & 7	56.94%	56.60%	57.50%	59.86%
Propensities: 1 & 8	57.86%	57.82%	59.79%	60.92%
Propensities: 2 & 3	56.60%	55.46%	60.29%	60.47%
Propensities: 2 & 4	55.45%	55.38%	56.71%	59.38%
Propensities: 2 & 5	55.22%	54.57%	55.64%	58.77%
Propensities: 2 & 6	57.12%	56.70%	58.21%	59.25%
Propensities: 2 & 7	56.76%	57.26%	56.14%	60.95%
Propensities: 2 & 8	57.28%	60.02%	61.71%	62.90%
Propensities: 3 & 4	55.34%	55.45%	58.50%	60.71%
Propensities: 3 & 5	56.26%	52.06%	57.50%	59.38%
Propensities: 3 & 6	54.82%	54.89%	56.57%	58.36%
Propensities: 3 & 7	53.84%	57.01%	56.64%	58.40%
Propensities: 3 & 8	57.57%	58.30%	59.21%	60.27%
Propensities: 4 & 5	52.64%	54.82%	53.71%	56.91%
Propensities: 4 & 6	54.36%	55.39%	57.07%	57.84%
Propensities: 4 & 7	56.71%	57.59%	55.29%	59.78%
Propensities: 4 & 8	58.03%	59.29%	62.07%	62.53%
Propensities: 5 & 6	53.33%	52.55%	54.14%	54.69%
Propensities: 5 & 7	55.85%	57.02%	57.50%	58.04%
Propensities: 5 & 8	58.32%	57.90%	60.29%	61.00%
Propensities: 6 & 7	57.11%	55.04%	56.14%	57.40%
Propensities: 6 & 8	56.99%	58.72%	58.07%	58.41%
Propensities: 7 & 8	57.39%	58.30%	60.29%	60.59%

At this point, an interesting question may arise: What would happen if instead of propensity we use amino acid identity representation, i.e., binary encoding of amino acids? Will binary encoding work equally well for B-cell epitope prediction? To assess the prediction power for amino acid identity representation, we have used binary coding for our data sets. In binary coding, each residue is encoded by a binary vector of length 20. Here each positive/negative sample with 20-mer is represented by a vector in 400 ( = 20×20) dimension. Hypothetically, if we number the 20 residues as 1 to 20, then code for the *i* ^th^ residue is a 1 vector of length 20 where all but the *i* ^th^ position is 1. The best prediction accuracies using a 2-level 10-fold cross validation scheme with SVM are 49.12%, 53.47%, 53.88%, and 52.79% when using amino acid identity representation (i.e., binary encoding) for data sets AAP872, ABCpred, BCPred, and Combo, respectively. Comparing these accuracies with those in Table 3, which are obtained using just a single propensity, we can infer that propensity-based encoding is far superior to binary encoding of amino acids for the purpose of epitope prediction. Note that, the dimension of binary-encoded data is 400, which is much higher than the dimension of the propensity-encoded data, which is just 20.

### Correlations of Paired Propensities

**Figure 1:**
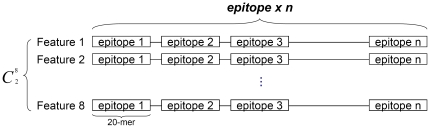
Encoding scheme for the calculation of correlations of pair amino acid propensities.

For both B-cell epitopes and non B-cell epitopes for each of four data sets, we check the correlations of paired propensities, as shown in the eight sub-tables of Table S1. To compute the correlation on say epitope data, first we concatenate all epitope fragments. Then to compute the correlation between the propensity pair (#*i*, #*j*), we create two sequences of values, one by replacing each residue by the corresponding value of propensity #*i* and the other by replacing each residue by the corresponding value of propensity #*j*. Then we compute the Pearson correlation coefficient between the two sequences. The encoding of peptides for computation of correlation is explained in Fig. 1. Table S1 also provides us with several interesting observations: (a) Irrespective of data sets and their types (B-cell or non B-cell epitopes), we consistently obtain similar correlation between pairs of propensities. Since for each of four data sets there is not much difference between correlation matrices for B-cell epitopes and non B-cell epitopes, it might be taken as an explanation of why these eight propensities do not contribute sufficient discriminating power for the epitope prediction (as shown in Table 5). (b) Two strongly correlated propensities together cannot add additional discriminating power, but two uncorrelated pair may. Table S1 shows that only in a few cases the correlation is very low. On the other hand, the very high correlation value between propensity #2 and propensity #4 suggests that the behaviour of these two attributes would be similar, together they may not add much and we have already seen that these are true.

### Conclusions

In this study, we have identified amino acid propensities that are good determinants of epitopes. We have also investigated the effectiveness of the important propensities in B-cell epitope prediction. In this context, we have used a novel group-feature selection neural network, GFSMLP, which exploits the interaction between propensities/features to select *groups* of useful propensities for improved linear B-cell epitope prediction. This system can also be used in other bioinformatics applications.

We have found that Chou's beta-turn (among the eight propensities considered) is the strongest determinant of the linear B-cell epitopes and that Chou's beta-turn can best cooperate with other propensities to yield better prediction performance, as well. In addition, we have seen that Chou's beta-turn collaborates together with Emini's Accessibility or Janin's Surface Exposed Scale to produce the best prediction results because each pair of propensities has a relatively lower correlation, respectively. Furthermore, we have observed that Emini's Accessibility and Janin's Surface Exposed Scale as expected have a very strong correlation and hence, as mentioned, they work well together with Chou's beta-turn in the same way. On the other hand, GFSMLP has also shown that neither Emini's Accessibility (a good determinant of linear B-cell epitopes) nor Janin's Surface Exposed Scale (*not* a good determinant of linear B-cell epitopes) cooperates with other propensities except with Chou's beta-turn. Note that, our primary objective is to find important determinants of epitopes not prediction accuracy. For this we have considered only eight propensities.

Our results confirm the utility of active feature selection, actually performed by GFSMLP, as opposed to the traditional passive approach of trying to use various combinations of amino acid propensities as input features without actually doing feature analysis. It opens up the possibility of applying GFSMLP to the more than 500 remaining amino acid propensities, and combinations thereof, and other features, to perform better B-cell epitope prediction. Developing bioinformatics analysis tools as a web service has become an inevitable trend nowadays [16]. Since the feature analysis task using the GFSMLP tool is computation-intensive, we do not provide a web server. But to help users with efficient analysis and to serve more users at the same time, we have developed the GFSMLP analysis tool as a stand-alone version with a friendly graphical user interface (GUI). The GUI version of GFSMLP program can be downloaded from: http://bio.classcloud.org/GFSMLP/.

## Methods

As suggested in [51], we first write the steps that one may follow in order to develop a useful predictor for an application: (i) Obtaining benchmark data sets to train and test the predictor; (ii) Mathematical formulation of prediction problem either explicitly (such as in regression) or implicitly (such as using neural networks) so that the model can capture the intrinsic relation hidden in the input-output data; (iii) Development of an efficient algorithm (or inference engine) to solve the prediction problem formulated in (ii); (iv) Performing cross-validation type tests to objectively evaluate the accuracy of the predictor; (v) Developing a stand-alone version with a friendly graphic user interface for the predictor and making that freely accessible. Basically, we follow these steps as explained in the subsequent section.

### Data Set Collection and Pre-processing

We obtain three B-cell epitope data sets from published literature, which are named as AAP872 [31], ABCpred [30] and BCPred [34]. A fourth set, named Combo, is generated combining these three sets. These data sets are subjected to some pre-processing as follows: First, for each of the three data sets, we remove redundant peptide sequences, respectively by our Perl script. If there are two or more sequences having 100% sequence identity, we just keep one of them. To remove the homologous sequences from the benchmark data sets, a cutoff threshold of 25% was imposed in [52] to exclude those proteins from the benchmark data sets that have more than 25% sequence identity to any other protein in the same subset. However, in this study we did not use this criterion in order to have similar data sets/computational protocol to compare prediction performance with other related previous studies. Thus, we keep the number of sequences the same as that in the original studies where possible. But the redundant sequences (sequences which are repeated more than once) and the problematic sequences (sequences with non-amino acid symbols or alphabets) are removed.

Then, we combine these three data sets into a new comprehensive data set named Combo. Again using our Perl script we remove the redundant peptide sequences from the Combo set. Note that, in each of the original three data sets, the positive samples (B-cell epitopes) and negative samples (non B-cell epitopes) are equal in number before the removal process. After the removal of redundant peptide sequences, each of the four sets may contain an unequal number of negative and positive samples. In the present case, for each data set we had more negative samples than positive ones. So we randomly remove negative samples from each of the four data sets to equalize the negative and positive samples. Also note that, the length of all the sequences in all of the data sets are 20 (a combined 20 kinds of amino acids) and finally for these four data sets, AAP872, ABCpred, BCPred, and Combo, there are 1,744, 1,228, 1,400, and 2,474 entries, respectively (Supplementary is also available at: http://bio.classcloud.org/GFSMLP/).

### Amino Acid Propensity and Sequence Encoding

Keeping consistency with previous studies [26], [27], [30], [31], [34], [35], we adopt eight widely used amino acid propensities as features for doing machine learning with the GFSMLP network [38]. These eight propensities are: (1) hydrophilicity [22], (2) accessibility [19], (3) flexibility [20], (4) surface exposed scales [40], (5) polarity [41], (6) turns [24], (7) antigenicity [23], and (8) beta-turn [42]. Each of these eight physico-chemical properties has 20 values for the 20 amino acids. We normalize these values between 1 and −1 as:
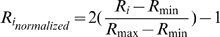
(1)where *R_max_* and *R_min_* represent the maximum and minimum values of the propensity. Then a residue is represented by 8 values, one for each of the 8 propensities. Since our sequence length is 20, the length of each encoded vector is 160 (20×8).

### Group Feature Selecting Multilayered Perceptron (GFSMLP)

For each of the four data sets, the epitope and non-epitope sequences are represented by a feature vector in 160 dimension. We can directly use these as a feature vector and design a machine learning system to predict epitopes and non-epitopes. But before that we want to raise a few questions. Are all of these propensities necessary to predict epitope and non-epitope? Which propensity has the strongest influence in determining epitope and non-epitope? These are biologically interesting questions. Moreover, if we can discard some propensity, this will also reduce the design cost and complexity of the decision making system. For this we cannot use usual feature selection mechanisms because the 160 features do not represent 160 different attributes, but they represent 8 groups of attributes. Thus if we reject one attribute, this will amount to rejecting 20 feature values relating to that attribute. This is a more complex feature selection problem, as it involves selection from among a set of groups of features. In bioinformatics, there are other similar problems. To address these issues, we use the GFSMLP network proposed in our previous study [38].

**Figure 2:**
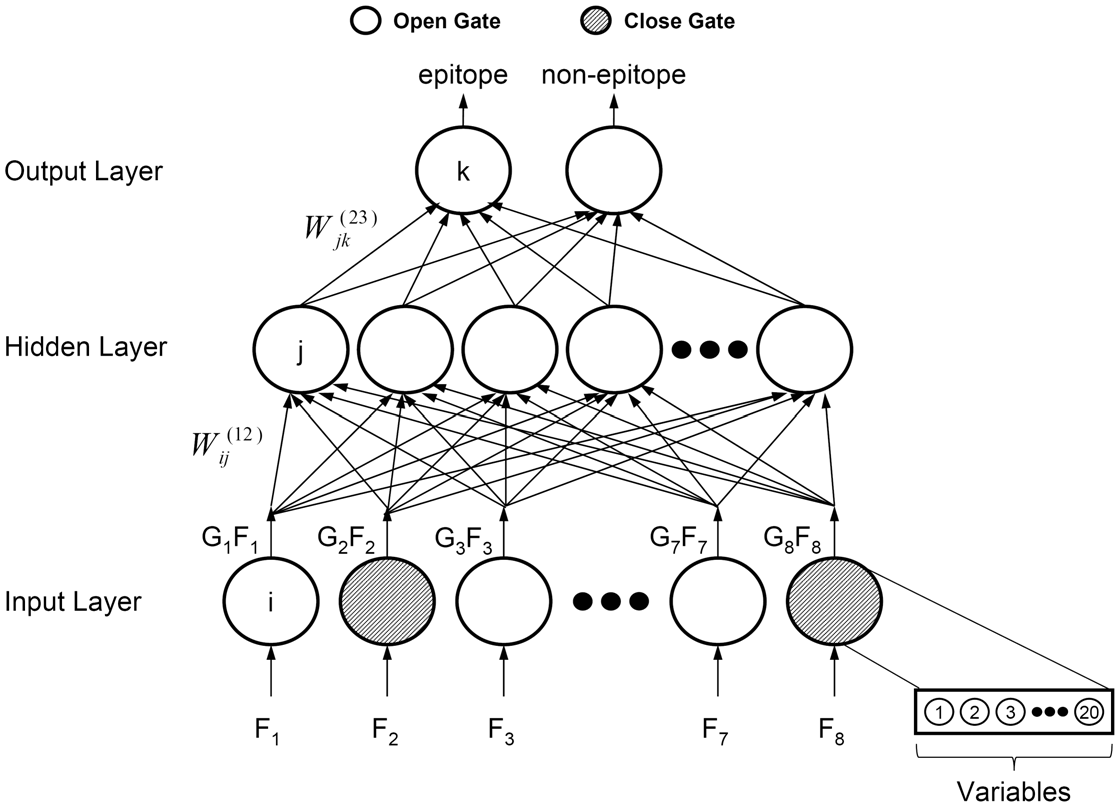
FSMLP network structure. Eight amino acid propensities are used in the input layer. Each propensity results in 20 normalized amino acid values. Thus the inputs are in 160-dimension. The 20 values corresponding to a particular propensity are treated as a group. The algorithm selects one or more propensities to evaluate its or their performance. After the training is over, the GFSMLP reports the most useful propensity/propensities to classify the input peptide sequence belonging to epitopes or non-epitopes in the output layer.

In Fig. 2, GFSMLP is shown as a three-layer network, including an input layer, hidden layer, and output layer. The GFSMLP can have more than one hidden layer, but we shall restrict to just one hidden layer. The input layer has 160 nodes, but the nodes are grouped in eight sub-groups, where each group has 20 values corresponding to a particular propensity. We associate an attenuator gate, *G_i_*, to each of these eight groups (or propensities); *G_i_* 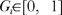
[0, 1]. Thus there will be eight gates. Each gate, *G_i_*, is mathematically modelled by a function with a tuneable parameter, λ_i_, which controls the opening and closing of the gates depending on its utility. Each feature *x_l_* of the *i* ^th^ group (*F_i_*) gets multiplied by the attenuator function *G_i_* before it gets into the network. Note that, *G_i_* = 0 means that the gate is closed and no feature of the *i* ^th^ group will get into the network. On the other hand, *G_i_* = 1 suggests that the associated gate is completely open and infers that every feature of the *i* ^th^ group enters the network unaltered. The objective of GFSMLP is to tune λ_i_ of *G_i_*(λ_i_) through the training process such that *G_i_*(λ_i_)→1, if the *i* ^th^ group of features is important and *G_i_*(λ_i_)→0, if the *i* ^th^ group is a *bad* group. At the beginning of training, all λ_i_s are so set that all *G_i_* values are nearly 0 (i.e., no amino acid propensity is important). Then after the training process, some of the *G_i_* values associated with useful amino acid propensities becomes close to 1. Details of the modelling and training can be found in [38].

For training the network, four parameters should be provided: the learning constant “*μ*” for the attenuator, the learning constant “*η*” for network's weights, the number of nodes “*n*” in the hidden layer, and the maximum number of iterations for each training process (here we refer to each training process as a run).

In this study, two kinds of experiments are performed to select good amino acid propensities. First, we make 1,000 GFSMLP runs and over these 1,000 runs we record the frequency with which different amino acid propensities are selected. Frequently occurring propensities are likely to be better discriminators of epitopes and non-epitopes when used separately. In other words, a propensity with a higher frequency suggests a better biological property than those with a lower frequency for prediction of epitope sites. As shown in Table 1, the eighth propensity “Beta-turn” is selected with the highest frequency for all four data sets and hence it can be considered the best biological property for the linear B-cell epitope prediction. The second experiment is designed to check whether any specific pair amino acid propensities will cooperate well together or not. Here at the onset of training, the gate corresponding to a particular amino acid propensity is set open, i.e., *G*(λ) = 1. If this is a bad attribute, then training will close this gate. If this is a good attribute and can cooperate with some other propensity, then the gate corresponding to the other propensity will be opened by the training. We repeat this experiment 100 times and count the frequency with which other gates are opened.

### A two-level 10-fold cross-validation scheme with SVM

Bootstrapping, Jackknifing and cross-validation are three similar statistical techniques that involve reuse of a given data set [53]. But, the purposes of this reuse of the samples are different for these three methods. Bootstrapping is used to evaluate the variance of an estimator while Jackknifing is used to reduce the bias of an estimator and to estimate the variance of an estimator. On the other hand, cross-validation is used to estimate the error involved in making predictions. It is also used for model selection. Yet researchers use all three methods for estimation of prediction error. Some authors prefer the Jackknifing method because the outcome obtained by the Jackknife test is always unique for a given benchmark data set as there is no randomness in selection of subsamples [54]–[60]. However, considering the present purposes, which involve both model selection and estimation of prediction error, we use here the cross-validation scheme.

**Figure 3:**
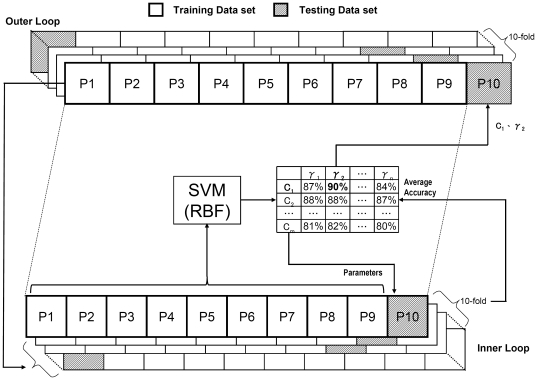
Scheme of two-level 10-fold cross-validation. The data set is partitioned into 10 parts (folds) in the outer loop. One fold of the data set is kept for testing of SVM. The remaining 9 folds are used as the training set for training an SVM. In the inner loop, the training set is further divided into 10 folds to choose the optimal parameters for testing the accuracy of the data set kept in the outer loop. The procedure is repeated 10 times.

We use the propensities selected by GFSMLP as the input information for the Support Vector Machine classifier [39]. So, as shown in Fig. 3, a two-level 10-fold cross-validation scheme with SVM is adopted to obtain an un-biased classification performance based on the selected propensities. Note that, in the outer level of the cross-validation scheme, we divide each data set into 10 parts (folds) of equal size (to the extent possible), and use 9 folds as a training set for training SVM and the remaining fold is kept for testing. In the inner level of the cross-validation scheme, the training set from the outer level is further divided into 10 folds to choose the optimal parameters for the SVM. The entire process is repeated 10 times, once for each of the 10 folds in the outer level, and the accuracy is recorded.

## Supporting Information

Table S1 **Correlation between each two propensities of all eight propensities.**
(DOC)
