# Correction: Identification of Amino Acid Propensities That Are Strong Determinants of Linear B-cell Epitope Using Neural Networks

**DOI:** 10.1371/annotation/58d3a7cb-d039-40ca-b960-bd60abfe0ae3

**Published:** 2012-03-22

**Authors:** Chun-Hung Su, Nikhil R. Pal, Ken-Li Lin, I-Fang Chung

There was an error in the funding statement. The correct funding statement is: "This work is supported in part by the National Science Council, Taiwan, under Contract NSC100-2319-B-010-002, and in part by the "A grant from Ministry of Education, Aim for the Top University Plan" of the National Yang-Ming University, Taiwan. The funders had no role in study design, data collection and analysis, decision to publish, or preparation of the manuscript." 

