# Supplementary material for: Identification of Amino Acid Propensities That Are Strong Determinants of Linear B-cell Epitope Using Neural Networks
Source: PLoS One. 2012 Feb 8;7(2):e30617. doi: 10.1371/journal.pone.0030617 (PMC3275595; doi:10.1371/journal.pone.0030617)
Supplement: Table S1 — Correlation between each two propensities of all eight propensities. (DOC) [file pone.0030617.s001.doc]

# Table S1.

| **AAP872 (B-cell epitopes)** | | | | | | | | |
| --- | --- | --- | --- | --- | --- | --- | --- | --- |
| **Propensities** | **#1** | **#2** | **#3** | **#4** | **#5** | **#6** | **#7** | **#8** |
| **#1** | 1.000 |  |  |  |  |  |  |  |
| **#2** | 0.694 | 1.000 |  |  |  |  |  |  |
| **#3** | 0.865 | 0.707 | 1.000 |  |  |  |  |  |
| **#4** | 0.568 | 0.955 | 0.636 | 1.000 |  |  |  |  |
| **#5** | 0.453 | 0.701 | 0.489 | 0.752 | 1.000 |  |  |  |
| **#6** | 0.447 | 0.246 | 0.315 | 0.139 | -0.053 | 1.000 |  |  |
| **#7** | -0.679 | -0.647 | -0.679 | -0.498 | -0.423 | -0.248 | 1.000 |  |
| **#8** | 0.639 | 0.393 | 0.612 | 0.249 | -0.007 | 0.650 | -0.510 | 1.000 |
| **AAP872 (non B-cell epitopes)** | | | | | | | | |
| **Propensities** | **#1** | **#2** | **#3** | **#4** | **#5** | **#6** | **#7** | **#8** |
| **#1** | 1.000 |  |  |  |  |  |  |  |
| **#2** | 0.728 | 1.000 |  |  |  |  |  |  |
| **#3** | 0.869 | 0.740 | 1.000 |  |  |  |  |  |
| **#4** | 0.604 | 0.955 | 0.672 | 1.000 |  |  |  |  |
| **#5** | 0.493 | 0.731 | 0.525 | 0.778 | 1.000 |  |  |  |
| **#6** | 0.458 | 0.255 | 0.335 | 0.138 | -0.006 | 1.000 |  |  |
| **#7** | -0.701 | -0.685 | -0.699 | -0.544 | -0.465 | -0.256 | 1.000 |  |
| **#8** | 0.680 | 0.449 | 0.650 | 0.305 | 0.069 | 0.664 | -0.538 | 1.000 |
| **ABCpred (B-cell epitopes)** | | | | | | | | |
| **Propensities** | **#1** | **#2** | **#3** | **#4** | **#5** | **#6** | **#7** | **#8** |
| **#1** | 1.000 |  |  |  |  |  |  |  |
| **#2** | 0.692 | 1.000 |  |  |  |  |  |  |
| **#3** | 0.865 | 0.711 | 1.000 |  |  |  |  |  |
| **#4** | 0.569 | 0.955 | 0.644 | 1.000 |  |  |  |  |
| **#5** | 0.455 | 0.703 | 0.500 | 0.753 | 1.000 |  |  |  |
| **#6** | 0.446 | 0.241 | 0.312 | 0.135 | -0.061 | 1.000 |  |  |
| **#7** | -0.673 | -0.646 | -0.675 | -0.500 | -0.426 | -0.249 | 1.000 |  |
| **#8** | 0.634 | 0.394 | 0.604 | 0.253 | -0.007 | 0.654 | -0.504 | 1.000 |
| **ABCpred (non B-cell epitopes)** | | | | | | | | |
| **Propensities** | **#1** | **#2** | **#3** | **#4** | **#5** | **#6** | **#7** | **#8** |
| **#1** | 1.000 |  |  |  |  |  |  |  |
| **#2** | 0.729 | 1.000 |  |  |  |  |  |  |
| **#3** | 0.864 | 0.739 | 1.000 |  |  |  |  |  |
| **#4** | 0.598 | 0.954 | 0.667 | 1.000 |  |  |  |  |
| **#5** | 0.479 | 0.721 | 0.515 | 0.771 | 1.000 |  |  |  |
| **#6** | 0.468 | 0.270 | 0.354 | 0.149 | -0.006 | 1.000 |  |  |
| **#7** | -0.712 | -0.681 | -0.704 | -0.538 | -0.458 | -0.276 | 1.000 |  |
| **#8** | 0.683 | 0.459 | 0.661 | 0.315 | 0.078 | 0.673 | -0.547 | 1.000 |
| **BCPred (B-cell epitopes)** | | | | | | | | |
| **Propensities** | **#1** | **#2** | **#3** | **#4** | **#5** | **#6** | **#7** | **#8** |
| **#1** | 1.000 |  |  |  |  |  |  |  |
| **#2** | 0.694 | 1.000 |  |  |  |  |  |  |
| **#3** | 0.864 | 0.704 | 1.000 |  |  |  |  |  |
| **#4** | 0.571 | 0.955 | 0.636 | 1.000 |  |  |  |  |
| **#5** | 0.457 | 0.702 | 0.488 | 0.752 | 1.000 |  |  |  |
| **#6** | 0.443 | 0.238 | 0.313 | 0.130 | -0.054 | 1.000 |  |  |
| **#7** | -0.678 | -0.650 | -0.676 | -0.503 | -0.427 | -0.245 | 1.000 |  |
| **#8** | 0.638 | 0.394 | 0.609 | 0.251 | -0.005 | 0.651 | -0.508 | 1.000 |
| **BCPred (non B-cell epitopes)** | | | | | | | | |
| **Propensities** | **#1** | **#2** | **#3** | **#4** | **#5** | **#6** | **#7** | **#8** |
| **#1** | 1.000 |  |  |  |  |  |  |  |
| **#2** | 0.723 | 1.000 |  |  |  |  |  |  |
| **#3** | 0.864 | 0.744 | 1.000 |  |  |  |  |  |
| **#4** | 0.602 | 0.956 | 0.677 | 1.000 |  |  |  |  |
| **#5** | 0.490 | 0.734 | 0.530 | 0.780 | 1.000 |  |  |  |
| **#6** | 0.450 | 0.242 | 0.328 | 0.129 | -0.010 | 1.000 |  |  |
| **#7** | -0.695 | -0.692 | -0.699 | -0.551 | -0.467 | -0.245 | 1.000 |  |
| **#8** | 0.683 | 0.446 | 0.654 | 0.309 | 0.078 | 0.663 | -0.529 | 1.000 |
| **Combo (B-cell epitopes)** | | | | | | | | |
| **Propensities** | **#1** | **#2** | **#3** | **#4** | **#5** | **#6** | **#7** | **#8** |
| **#1** | 1.000 |  |  |  |  |  |  |  |
| **#2** | 0.693 | 1.000 |  |  |  |  |  |  |
| **#3** | 0.864 | 0.710 | 1.000 |  |  |  |  |  |
| **#4** | 0.568 | 0.955 | 0.639 | 1.000 |  |  |  |  |
| **#5** | 0.452 | 0.702 | 0.491 | 0.751 | 1.000 |  |  |  |
| **#6** | 0.446 | 0.243 | 0.314 | 0.137 | -0.056 | 1.000 |  |  |
| **#7** | -0.677 | -0.647 | -0.677 | -0.500 | -0.425 | -0.245 | 1.000 |  |
| **#8** | 0.636 | 0.394 | 0.610 | 0.252 | -0.008 | 0.652 | -0.504 | 1.000 |
| **Combo (non B-cell epitopes)** | | | | | | | | |
| **Propensities** | **#1** | **#2** | **#3** | **#4** | **#5** | **#6** | **#7** | **#8** |
| **#1** | 1.000 |  |  |  |  |  |  |  |
| **#2** | 0.727 | 1.000 |  |  |  |  |  |  |
| **#3** | 0.866 | 0.743 | 1.000 |  |  |  |  |  |
| **#4** | 0.602 | 0.955 | 0.674 | 1.000 |  |  |  |  |
| **#5** | 0.492 | 0.731 | 0.525 | 0.777 | 1.000 |  |  |  |
| **#6** | 0.458 | 0.259 | 0.338 | 0.142 | 0.005 | 1.000 |  |  |
| **#7** | -0.700 | -0.685 | -0.698 | -0.544 | -0.465 | -0.253 | 1.000 |  |
| **#8** | 0.680 | 0.454 | 0.655 | 0.313 | 0.079 | 0.665 | -0.535 | 1.000 |
